# Therapeutic Evaluation of *Bifidobacterium animalis* subsp. *lactis* MH-02 as an Adjunctive Treatment in Patients with Reflux Esophagitis: A Randomized, Double-Blind, Placebo-Controlled Trial

**DOI:** 10.3390/nu16030342

**Published:** 2024-01-24

**Authors:** Lihong Gan, Yufan Wang, Shenan Huang, Li Zheng, Qi Feng, Hui Liu, Peng Liu, Kaige Zhang, Tingtao Chen, Nian Fang

**Affiliations:** 1 Third Clinical Medical College, Jiangxi Medical College, Nanchang University, Nanchang 330006, China; 2 Department of Gastroenterology, The First Hospital of Nanchang, Nanchang 330006, China; 3 Queen Mary School, Nanchang University, Nanchang 330031, China; 4 Department of Gastrointestinal, The Second Affiliated Hospital of Nanchang University, Nanchang 330006, China; 5 National Engineering Research Center for Bioengineering Drugs and the Technologies, Institute of Translational Medicine, Nanchang University, Nanchang 330036, China; 6 School of Pharmacy, Jiangxi Medical College, Nanchang University, Nanchang 330006, China

**Keywords:** *Bifidobacterium animalis* subsp. *lactis* MH-02, RE, PPI, reflux, gastrointestinal symptoms, recrudescence, gut microbiota

## Abstract

Proton pump inhibitors (PPIs) are currently routinely used for the treatment of reflux esophagitis (RE); however, with frequent symptom recurrence after discontinuation and limited clinical improvement in accompanying gastrointestinal symptoms. This study aims to explore the adjuvant therapeutic effect of *Bifidobacterium* supplement for RE patients. A total of 110 eligible RE patients were recruited and randomly assigned to the placebo and probiotic groups. All patients were treated with rabeprazole tablets and simultaneously received either *Bifidobacterium animalis* subsp. *lactis* MH-02 or placebo for 8 weeks. Patients who achieved clinical remission then entered the next 12 weeks of follow-up. RDQ, GSRS scores, and endoscopy were performed to assess clinical improvement, and changes in intestinal microbiota were analyzed with high-throughput sequencing. Our results revealed that MH-02 combined therapy demonstrated an earlier time to symptom resolution (50.98% vs. 30.61%, *p* = 0.044), a significant reduction in the GSRS score (*p* = 0.0007), and a longer mean time to relapse (*p* = 0.0013). In addition, high-throughput analyses showed that MH-02 combined therapy increased the α (*p* = 0.001) diversity of gut microbiota and altered microbial composition by beta diversity analysis, accompanied with significantly altered gut microbiota taxa at the genus level, where the abundance of some microbial genera including *Bifidobacterium*, *Clostridium,* and *Blautia* were increased, while the relative abundance of *Streptococcus* and *Rothia* were decreased (*p* < 0.05). Collectively, these results support the beneficial effects of MH-02 as a novel complementary strategy in RE routine treatment.

## 1. Introduction

Reflux esophagitis (RE) is a primary subtype of gastroesophageal reflux disease (GERD), which is a prevalent digestive system disorder worldwide [1,2]. The initial symptoms of RE include acid reflux and heartburn, while severe cases can potentially progress to complications like esophageal narrowing, bleeding, and even Barrett’s esophagus [3]. Currently, proton pump inhibitors (PPIs) are the first-line medications for treating RE, providing relief for approximately 80% of patients’ primary symptoms [4]. However, PPIs have limited efficacy against accompanied digestive symptoms like upper abdominal pain and bloating, and symptoms often recur after discontinuation of treatment. Consequently, actively seeking new complementary approaches in RE management has become an urgent concern for clinicians.

The pathogenesis of RE is intricate, encompassing a wide range of risk factors including weakened esophageal clearance, impaired mucosal barrier function, and inflammatory-mediated effects [5]. Accumulating evidence has now revealed that dysbiosis in the gut microbiota may contribute to the onset and further progression of RE, and the manifested reduced microbial diversity and abundance in intestine of individuals with GERD have been reported when compared to healthy counterparts [6]. Moreover, RE patients demonstrated a disparate microbiota profile in oral, esophagus, and gut compared with healthy individuals which were dominated by taxa refluxing from oropharynx [7]. Further, both in vitro and in vivo experiments have demonstrated that modifications in certain esophageal microbiomes can aggravate inflammation and increase the risk of reflux and metaplasia in the esophagus by directly eliciting a pro-inflammatory response and up-regulating lipopolysaccharide (LPS) signaling, suggesting microbiota in the digestive tract may serve an instrumental role in initiation and progression of RE [8,9,10]. However, present therapeutic recommendations included PPIs which have been reported to possibly elicit dysbacteriosis [11]. And, long-term application of PPIs can further induce small intestinal bacterial overgrowth (SIBO), which can contribute to an ongoing inflammatory reaction, decrease responsiveness of esophageal smooth muscle cells, and increase the production of methane, ultimately favoring the backflow of stomach contents into the esophagus [12,13]. Therefore, interventions targeting the gut microbiota may represent a novel therapeutic approach for the treatment of reflux esophagitis.

At present, probiotic supplementation as a predominant intervention method for modulation of gut microecology, has been extensively utilized in prevention and treatment of human gastrointestinal (GI) microbiome-related diseases [14,15,16]. *Bifidobacterium*, a crucial member of GI probiotics, confers physiological benefits via diverse mechanisms, including disposing metabolites, alleviating inflammatory response, and manipulating immune function [17,18]. A recent clinical trial involving 20 pregnant women demonstrated that administration of a probiotic complex containing *Bifidobacterium* can effectively reduce reflux episodes [19]. And, some other reports also suggested that the supplement of fermented milk containing *Bifidobacterium bifidus* can alleviate gastrointestinal discomforts including abdominal pain, bloating, and diarrhea in individuals with functional digestive disorders [20,21,22]. Furthermore, our prior studies have also discovered that oral administration of *Bifidobacterium* can not only ameliorate gastrointestinal symptoms in patients’ post-gastrointestinal surgery, but also can contribute to the mitigation of inflammation and restoration of intestinal microbiota diversity [23,24]. Nevertheless, it is uncertain whether the combination of *Bifidobacterium* with PPIs can exert a synergistic effect in enhancing therapeutic effectiveness in managing RE.

Therefore, here, we designed a prospective clinical study aiming at evaluating the efficacy of *Bifidobacterium animalis* subsp. *lactis* MH-02 to improve the clinical treatment in patients with RE. Additionally, we also performed high-throughput sequencing to evaluate the alternations in gut microbiota following MH-02 intervention. This study may provide evidence-based support for the use of probiotics in the adjunctive treatment of RE.

## 2. Materials and Methods

### 2.1. Ethics Statement

All the patients participating in this study signed the informed consent form. All the procedures in this study were reviewed and approved by the Ethics Committee of the Nanchang First Hospital (KY2021071), and the study was registered in the Chinese Clinical Trial Registry with the registration number ChiCTR2200059624. Our clinical trials strictly adhered to the following laws or guidelines: the Declaration of Helsinki, the international ethical guidelines of the World Health Organization (WHO), and the China Good Clinical Practice Guidelines (Issue 57, 2020).

### 2.2. Study Design and Participants Selection Criteria

This study was a single-center, randomized, double-blind, placebo-controlled trial. Through June 2022 to June 2023, outpatient or inpatient patients receiving treatment in the Department of Gastroenterology at the Nanchang First Hospital were recruited, and all patients had complete clinical records. The diagnosis of RE was based on the 2013 guidelines for diagnosis and management of reflux esophagitis [25]. The inclusion criteria were as follows: (1) confirmed diagnosis of reflux esophagitis (including Los Angeles classification grades LA, LB, LC) through clinical presentation and gastroscopy; (2) an age of 18–65 years; (3) no prior use of PPIs, or cessation of PPIs treatment for at least 1 month; and (4) no use of antibiotics, probiotics, lactulose, other antacids, or prokinetic drugs in the past 4 weeks. The exclusion criteria were as follows: (1) history of cirrhosis, renal impairment, inflammatory bowel disease, tumors, thyroid disorders, diabetes, severe cardiovascular, and cerebrovascular diseases; (2) digestive ulcers, gastrointestinal bleeding, esophageal stenosis, chronic diarrhea, or constipation, malabsorption; (3) History of gastrointestinal surgery; (4) pregnant or breastfeeding women; (5) patients undergoing immunosuppressive therapy; (6) allergy to rabeprazole, probiotics, or placebo components used in the study; and (7) participation in other drug clinical trials in the past three months. The First Hospital of Nanchang was responsible for collecting all clinical data.

### 2.3. Randomization and Blinding

Participants were allocated in a 1:1 ratio to either the probiotic group (Probio) or the placebo group using a random number table method. A non-participating trial staff labeled the corresponding probiotics and placebo with random numbers. Then, dedicated medication management staff provided the participants with the probiotics agent and placebo corresponding to their random number. Additionally, there were no significant differences in packaging, color, or odor between the probiotics and placebos to ensure the concealment of allocation. Throughout the study, the researchers who were responsible for sample distribution, data collection, data organization, and analysis, as well as the participants, remained unaware of the randomization sequence. The blinding was only disclosed in case of significant adverse events.

### 2.4. Gastroscopy and Grading of Esophagitis 

All gastroscopy examinations were conducted by two experienced senior physicians following a standardized protocol. Endoscopic assessment was performed at baseline for all patients enrolled and again at end of treatment (week 8). Esophagitis grade was classified based on the results of gastroscopy using the Los Angeles Classification grading system (Grade A: <5 mm mucosal breaks; Grade B: mucosal breaks >5 mm; Grade C: mucosal breaks extending between the tops of two mucosal folds but <75% of the circumference; Grade D: mucosal breaks extending >75% of the circumference). Improvement in esophagitis grading was calculated based on following criteria: (1) healing: improvement to grade N (normal); (2) significant improvement: improved by 2 grades; (3) effective improvement: improved by 1 grade; and (4) ineffective improvement: no change.

### 2.5. Reflux Disease Questionnaire (RDQ) and Gastrointestinal Symptom Rating Scale (GSRS)

A Reflux Disease Questionnaire (RDQ) was used to assess subjective reflux symptoms over a 1-week recall period. RDQ included four symptoms, including heartburn, chest pain, acid regurgitation, and food regurgitation. Symptoms were rated on a scale of 0–5 based on frequency and severity. The total score ranged from 0 to 40, with higher scores indicating more severe symptoms. Patients with an RDQ score ≥ 12 were considered to be either not in remission or with recurrence. The Gastrointestinal Symptom Rating Scale (GSRS) is a self-assessment scale comprising 15 items categorized into 5 main symptom clusters: abdominal pain (abdominal pain, hunger pain, and nausea); reflux syndrome (burning sensation and acid regurgitation); diarrhea syndrome (diarrhea, loose stools, and urgency); dyspeptic syndrome (abdominal distention, bloating, eructation, and increased flatulence); and constipation syndrome (constipation, hard stools, and sense of difficulty in defecation). Each item was scored on a Likert scale of 0–3 (none, mild, moderate, severe) based on the severity of symptoms over the past week. The total score ranged from 0 to 45, with higher scores indicating more severe symptoms. To minimize rater variability, all scoring was performed by the same physician.

### 2.6. Clinical Intervention Strategy and Management 

Stage 1: Patients who met the criteria were randomly assigned to either probiotics (Probio) or placebo (Placebo). Probiotics and placebo were provided by Heilongjiang Meihua Biotechnology Co, Ltd (Harbin, China), and stored in a refrigerator at 4 °C. Experimental probiotics, a mixture of *Bifidobacterium animalis* subsp. *lactis* MH-02 (CGMCC No.2899) and maltodextrin, contained 2 × 10^9^ colony-forming units (CFU) per package of viable bacteria. The placebo agent only contained maltodextrin in the same quantity as the probiotics agent and was identical in appearance, size, and color to the active intervention. Rabeprazole sodium enteric-coated tablets, 10 mg/tablet, were provided by Eisai (China) Pharmaceutical Co, Ltd (Suzhou, China). Patients in both the probiotics group and Placebo group were all administered with rabeprazole sodium enteric-coated tablets (1 tablet BID), while the probiotics group received a sachet of *Bifidobacterium* MH-02 (once daily) and the Placebo group received a sachet of placebo (once daily), both for 8 weeks. Drug compliance (PPI and probiotics/placebo) was assessed through biweekly telephone inquiries and by calculating the number of medication sachets consumed. Poor compliance was defined as missing a dose for ≥3 days. During this period, GSRS and RDQ scores were collected at baseline and weeks 1–8 (once a week), to evaluate patients’ improvement in clinical symptoms. An endoscopy was conducted at week 8 to evaluate esophageal mucosal improvement or healing. Additionally, stool samples were collected before and 8 weeks after intervention.

Stage 2: Patients who achieved both endoscopic (complete esophageal mucosal healing) and clinical (RDQ < 12) remission in stage 1 were then continued follow-up. The follow-up endpoint was defined as symptomatic relapse (RDQ ≥ 12) or the end of the 12-week follow-up period (week 20). During the follow-up period, all subjects underwent biweekly telephone inquiry or clinical follow-up, to complete GSRS and RDQ assessments.

### 2.7. Adverse Events and Prohibited Medications 

Adverse events were monitored throughout the study. Patients were prohibited from consuming any other probiotics or prebiotics and were instructed to continue their usual diet and lifestyle habits. During the follow-up period, discontinuation of acid-suppressing agents, prokinetic agents, or other drugs that might affect study outcomes was allowed only in cases of symptom relapse or the emergence of other symptoms requiring relevant medication. Concurrent use of drugs that did not affect study outcomes was permitted, with records of their drug intake.

### 2.8. DNA Extraction and High-Throughput Sequencing

Total microbial genomic DNA were extracted using TIANamp Stoll DNA Kit (TIANGEN Biotech Co., Ltd., Beijing, China; Catalog No.: DP328) following the manufacturer’s instructions, and stored at −20 °C prior to further analysis. The quantity and quality of extracted DNA was measured using a NanoDrop NC2000 spectrophotometer (Thermo Fisher Scientific, Waltham, MA, USA) and agarose gel electrophoresis, respectively. The subsequent PCR amplification of the V4 region of 16S rRNA (520F 5′-AYTGGGYDTAAAGNG-3′, 802R 5′-TACNVGGGTATCTAATCC-3′), sequencing library construction, and high-throughput sequencing (Illumina NovaSeq 6000 platform) were performed at Shanghai Personalbio Technology Co., Ltd. (Shanghai, China). Microbiome bioinformatics were performed with QIIME2 2019.4 and sequences were then quality filtered, denoised, merged, and chimera removed using the DADA2 plugin. The Greengenes database v13.8 (19) was used for taxonomy classification. High-throughput sequencing data of this trial have been uploaded to the NCBI database (PRJNA1036777). 

### 2.9. Results Evaluation

The primary outcomes were evaluated post-treatment using RDQ, GSRS, and endoscopy assessments, including overall cure rate, time to achieve primary symptom relief, improvement in accompanying gastrointestinal symptoms, and improvement in endoscopic esophagitis grade. Secondary outcomes included two aspects: firstly, evaluating patient relapse through RDQ and GSRS scores after the follow-up period; secondly, based on fecal samples, comparing changes in gut microbiota between groups, including differences in alpha diversity, beta diversity, and species composition.

### 2.10. Data Analysis

Clinical data were analyzed and charted using GraphPad Prism (v8.0.2) software. Quantitative data were presented as mean ± standard deviation or median (interquartile range), while qualitative data were presented as ratios. Non-paired *t*-tests or non-parametric Mann–Whitney tests were used for quantitative data, and Fisher’s exact test or Chi-square test for qualitative data. Two-sided *p* < 0.05 was considered statistically significant. Kaplan–Meier analysis was used to assess the cumulative relapse rate of RE. Microbiota analysis of fecal samples was conducted using the QIIME2(2019.4) software package and GraphPad Prism (v8.0.2) software for calculation and visualization. The α-diversity test was conducted using the Kruskal–Wallis rank-sum test and Dunn’s test as a post hoc test, β-diversity was assessed based on Jaccard and unweighted UniFrac distances and species relative abundances were compared by one-way analysis of variance (ANOVA) followed by the Kruskal–Wallis non-parametric test.

## 3. Results

### 3.1. Inclusion of Patients and Clinical Baseline Characteristics

**Figure 1:**
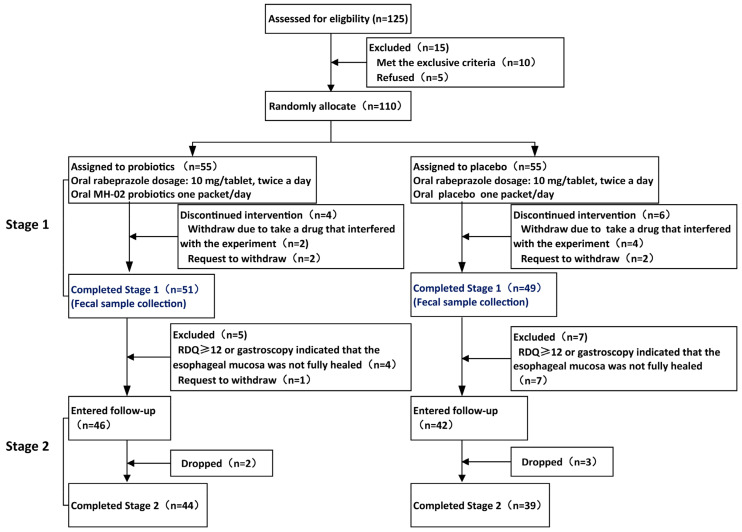
Flowchart of the whole trial. RDQ: Reflux diagnostic questionnaire.

Patient Enrollment and Clinical Baseline Characteristics: In stage 1, a total of 110 eligible patients were enrolled, with 55 randomized assigned to probiotics group (Probio) and 55 to placebo group (Placebo). Among them, four patients in the Probio group and six patients in the Placebo group did not complete the intervention due to taking medications that could potentially affect the experimental results during the treatment or requested withdrawal. The first phase of treatment was completed by 51 individuals in the probiotics group and 49 in the Placebo group. Subsequently, based on symptom scoring and endoscopic evaluation, 46 and 42 people, respectively, met the criteria to enter the second stage. During the follow-up period, 2 participants in the Probio group and 3 in the Placebo group dropped out, leaving a final total of 44 individuals in the probiotics group and 39 in the placebo group who completed the second phase of the study (see whole experimental schedule in Figure 1). Baseline characteristics and questionnaire scores of the patients are presented in Table 1. At baseline, there were no statistically significant differences between the two groups in terms of age, gender, body mass index (BMI), smoking history, anxiety and depression symptoms, esophagitis grading, or GSRS and RDQ scores (*p* > 0.05). Both groups exhibited balanced overall status, ensuring the comparability of the experimental results.

### 3.2. Combining of Probiotics Can Reduce Symptom Relief Time and Alleviate Gastrointestinal Symptoms in Patients with RE

**Figure 2:**
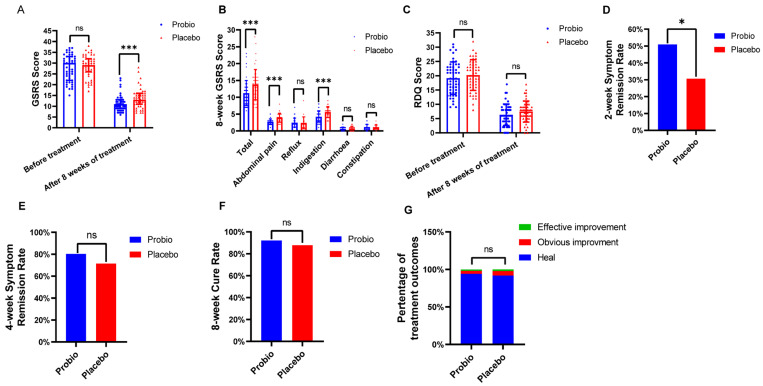
**Evaluation of clinical effect after intervention with probiotics.** (**A**) GSRS score: before treatment and after 8 weeks of treatment. (**B**) Symptom group score of GSRS score. (**C**) RDQ score: before treatment and after 8 weeks of treatment. (**D**) 2−week symptom remission rate. (**E**) 4−week symptom remission rate. (**F**) 8−week cure rate. (**G**) Comparison of esophagitis grade improvement. Probio: Oral rabeprazole and MH−02; Placebo: Oral rabeprazole and placebo. GSRS: Gastrointestinal Symptom Rating Scale. *: *p* < 0.05, ***: *p* < 0.001, ns: not significant.

We enrolled 110 individuals in the first stage of the trial, and the final 100 completed the required 8-week intervention. The RDQ score, GSRS score, time of relief in major symptom, and cure rate after treatment are shown in Figure 2. After 8 weeks of intervention, in Placebo group, the RDQ total score was 7.45 ± 3.68, and the GSRS total score was 13 (11, 16); however, when supplemented with MH-02, they were 6.33 ± 3.74 and 11 (9, 13), respectively. There was a significant decrement of GSRS score in the Probio group relative to the Placebo group (*p* = 0.0007) (Figure 2A), although there was no statistically significant difference in RDQ score (*p* = 0.136) (Figure 2C), indicating that combination MH-01 with rabeprazole could assist in improving the concomitant gastrointestinal symptoms in patients with RE. In the GSRS total score, the scores of abdominal pain (2.73 ± 0.83 vs. 3.93 ± 1.33, *p* < 0.001) and dyspepsia syndrome (4.22 ± 1.62 vs. 5.61 ± 1.63, *p* < 0.001) decreased significantly, while reflux syndrome, diarrhea syndrome, and constipation syndrome scores were not significantly different from the placebo group (Figure 2B). Observing the time to achieve primary symptoms relief in both groups, it was found that the improvement rate in Probio group within first 2 weeks (50.98%) was significantly higher than that in the Placebo group (30.61%), with a statistically significant difference (*p* = 0.044) (Figure 2D). However, no significant difference was observed in the improvement rate of primary symptoms between the two groups at week 4 (80.39% vs. 71.43%, *p* = 0.353) (Figure 2E). Furthermore, the cure rate at week 8 (92.16% vs. 87.76%, *p* = 0.689) (Figure 2F) also showed no statistically significant difference. Finally, by assessing the improvement in esophagitis grading based on follow-up endoscopy results, the endoscopic healing rate of esophageal mucosa in both groups reached more than 90% (Figure 2G), with no statistically significant difference between the two groups. These results indicate that even though probiotics combined application exerted no notable impact on the ultimate cure rate or endoscopic healing, it can shorten the time to achieve primary symptoms relief and improve gastrointestinal symptoms in patients.

### 3.3. Combining Probiotics Therapy Can Delay the Recurrence of Symptoms

Upon completion of the first stage of treatment, the patients were assessed by RDQ scores and esophageal mucosal healing by endoscopic examination. Patients who achieved an RDQ score below 12 and completed esophageal mucosal healing were considered cured. In this study, a total of 88 cured patients entered the second stage of follow-up, with 46 patients in the probiotics group and 42 in the Placebo group. Finally, 42 individuals in the probiotics group and 39 individuals in the Placebo group completed the entire trial.

**Figure 3:**
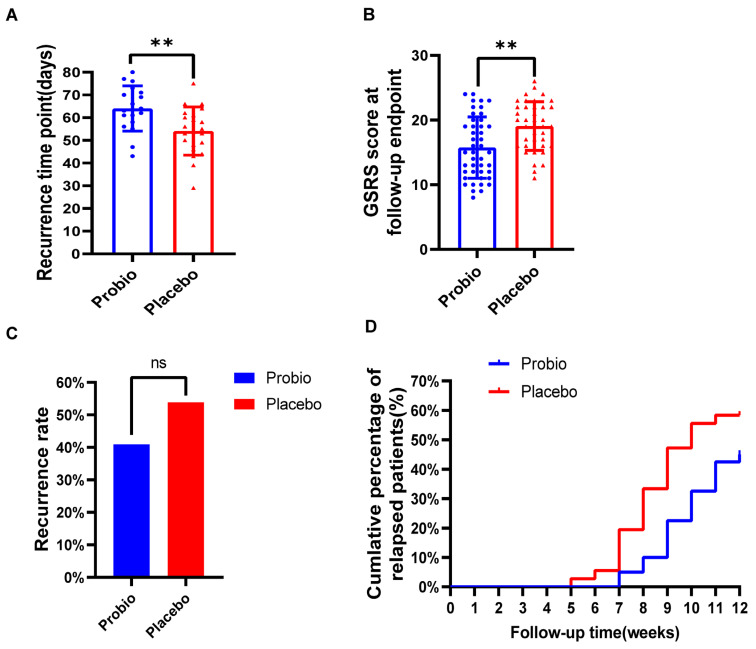
**The combination of probiotics delayed the recurrence of symptoms in RE patients.** (**A**) Recurrence time of symptoms. (**B**) GSRS scores at follow-up endpoint. (**C**) Overall recurrence rate in both groups. (**D**) The cumulative recurrence rate of patients. Probio: Oral rabeprazole and MH−02; Placebo: Oral rabeprazole and placebo. **: *p* < 0.01, ns: no significant.

Among 88 eligible cured patients, 44 patients in the Probio group and 39 patients in the Placebo group completed the subsequent 12-week follow-up. At the follow-up endpoint, 18 patients in the Probio group experienced recurrence, with an average recurrence time of 64.00 ± 9.99 days, while 21 patients in the Placebo group experienced recurrence, with a shortened average recurrence time of 54.10 ± 10.58 days. Although there was no significant difference in the overall recurrence rate between the two groups (40.91% vs. 53.85%, *p* = 0.275) (Figure 3C), there was a significant prolonged recurrence time (*p* = 0.005) in the Probio group comparing to Placebo group (Figure 3A). Furthermore, the GSRS total score in the Probio group was significantly lower than that in the Placebo group (15(12, 19) vs. 19(16, 22), *p* = 0.0013), as demonstrated by the statistical difference depicted in Figure 3B. And, as shown in Figure 3D, the cumulative recurrence rate curve indicated that the recurrence time in the Placebo group was earlier than that in the Probio group, and moreover, the cumulative recurrence rate in the Placebo group tended to be higher than that in the Probio group almost every week; for example, at week 8 (9.1% vs. 30.8%) and week 10 (29.5% vs. 51.3%).

### 3.4. Combined Probiotics Therapy Contributes to Enrich the Diversity of Gut Microbiota

In total, we collected 150 stool samples, including 50 samples collected before treatment (RE) and 100 samples (51 from the Probio and 49 from the Placebo) collected after the completion of the first phase of trial. 

**Figure 4:**
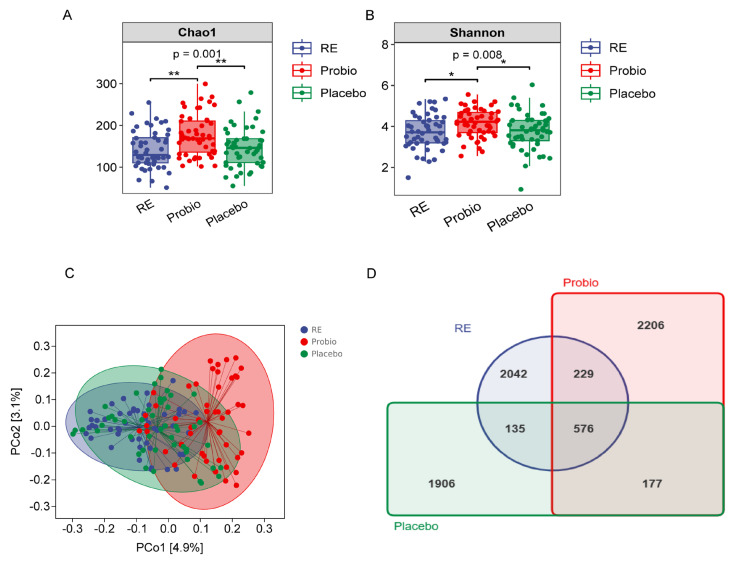
**Probiotics increased the diversity of the gut microbiota in patients with RE.** (**A**) The Chao1 index. (**B**) The Shannon index. (**C**) PCoA of β−diversity index based on the Jaccard distance. (**D**) Venn diagram of the identified bacterial species. RE: Reflux esophagitis before treatment. Probio: Oral rabeprazole and MH−02; Placebo: Oral rabeprazole and placebo. *: *p* < 0.05, **: *p* < 0.01.

In the α-diversity analysis, the Chao1 and Shannon indices in the Probio group significantly increased compared with RE and Placebo groups (*p* < 0.01) (Figure 4A,B). However, there was no significant difference in the two indices between the Placebo group and the RE group. The trend in the observed species index was similar to that of the Chao1 index that was higher in the Probio group relative to the two other groups (Supplementary Figure S1A), and the average Goods coverage rate for each group’s samples was above 99.9% (Supplementary Figure S1B). Additionally, the sparse curve in Supplementary Figure S1C indicated that the sequencing data volume in this experiment was sufficient.

In the β-diversity analysis, the analytic results from PCoA, which was based on Jaccard and weighted UniFrac distance matrices, revealed that the majority of samples in the Probio group were notably distant from those in the RE and Placebo groups, while most samples from the RE and Placebo groups overlapped with each other (Figure 4C, Supplementary Figure S1D). Furthermore, after quality control, a total of 8975 operational taxonomic units (OTUs) were generated from the sequenced samples, with 576 OTUs shared among all groups. The order of OTUs or total fecal bacteria count in each group was as follows: Probio > RE > Placebo (Figure 4D). These results indicate that combined probiotics therapy in RE patients contributes to an increase in the diversity of their gut microbiota.

### 3.5. Combined Probiotics Therapy Altered the Composition of the Intestinal Microbiota

**Figure 5:**
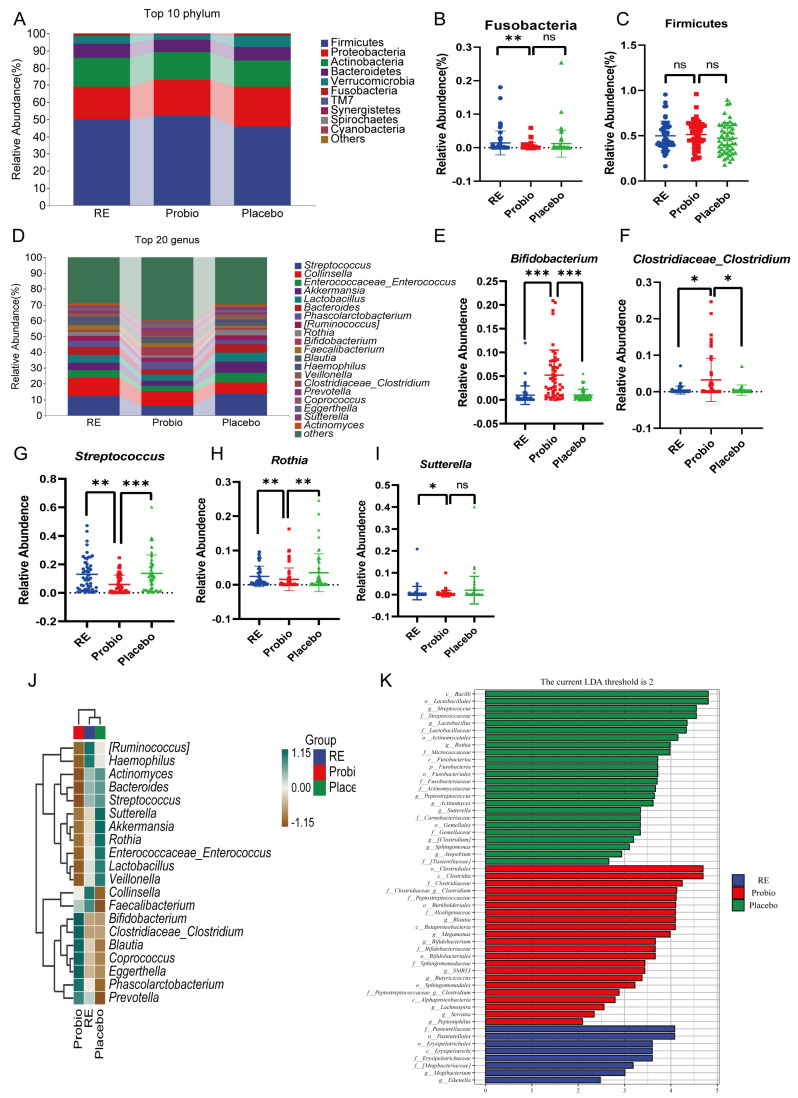
**Probiotics modulate the composition of gut microbiota in patients with RE.** (**A**) Species composition analysis map at the phylum level. (**B**,**C**) The relative abundance of Fusobacteria and Firmicutes. (**D**) Species composition analysis map at the genus level. (**E**–**I**) The relative abundance of *Bifidobacterium*, *Clostridiaceae_Clostridium*, *Streptococcus*, *Rothia,* and *Sutterella*. (**J**) Clustering heat map based on the top 20 positions of the genus level. (**K**) Histogram of LDA value distribution (LDA threshold score > 2). RE: Reflux esophagitis before treatment. Probio: Oral rabeprazole and MH−02; Placebo: Oral rabeprazole and placebo. *: *p* < 0.05, **: *p* < 0.01, ***: *p* < 0.001, ns: no significant.

Across all groups, bacterial phyla including Firmicutes, Bacteroidetes, Actinobacteria, Proteobacteria, and Verrucomicrobia collectively constituted more than 90% of the microbial composition (Figure 5A). Further statistical analysis revealed that the abundance of Fusobacteria at the phylum level was significantly reduced in the Probio group compared to the RE group (*p* < 0.05) (Figure 5B). And, there was also a slight increase in Firmicutes, and a decrease in Proteobacteria, Bacteroidetes, and Verrucomicrobia in the Probio group compared to the Placebo group (Figure 5C, Supplementary Figure S2A–C). For the top 20 genus level (Figure 5D), it was observed that the relative abundance of *Bifidobacterium* and *Clostridiaceae_Clostridium* was significantly higher in the Probio group compared to both the RE and Placebo groups (Figure 5E,F). Conversely, the relative abundance of Streptococcus and Rothia was significantly lower in the Probio group compared to the RE and Placebo groups (Figure 5G,H). And, the relative abundance of *Sutterella* was also lower in the Probio group compared to the RE group (Figure 5I). These findings indicate that combined probiotics therapy for RE leads to significant changes for the composition of gut microbiota at the genus level.

A clustering heatmap was generated based on the average abundance of the top 20 species at the genus level (Figure 5J). We can observe that certain beneficial gut bacteria have higher relative abundance in the Probio group compared to the other groups, such as *Bifidobacterium*, *Clostridiaceae_Clostridium*, *Blautia*, *Coprococcus*, *Phascolarctobacterium*, and *Prevotella*. On the other hand, bacteria like *Akkermansia*, which is involved in mucin degradation, *Sutterella*, associated with intestinal inflammation, and common potentially pathogenic genera like *Enterococcus* and *Streptococcus*, were more enriched in the Placebo group compared with the Probio group. Of note, *Lactobacillus*, which is generally considered beneficial, had a higher abundance in the Placebo group and a lower abundance in the Probio group which may be due to the use of proton pump inhibitors (PPIs) in this study. And, previous research has supported that the use of PPIs can significantly increase *Lactobacillus* levels and disrupt the ecological structure of the other gut microbiota [11,26,27]. While in this study, probiotics treatment may have corrected this imbalance. LEfSe analysis (≥2) also revealed the high abundance bacterial taxa that were majorly different in fecal samples among the different groups (Figure 5K and Supplementary Figure S3). In the Probio group, beneficial taxa such as *Bifidobacterium* and short-chain fatty acid-producing bacteria like *Blautia*, *Butyricicoccus*, *Lachnospira*, and *Clostridium* were more enriched relative to the other groups. In contrast, the Placebo group exhibited enrichment of taxa from *Streptococcaceae* to *Streptococcus*, *Enterococcaceae* to *Enterococcus*, *Actinomycetaceae* to *Actinomyces*, *Lactobacillaceae* to *Lactobacillus*, *Rothia*, and *Sutterella*. In summary, the above results indicate that the combined use of probiotics may play a positive role in promoting the growth of beneficial gut bacteria and restrain the growth of potentially pathogenic bacteria.

### 3.6. Probiotics Supplement Can Potentially Reduce Adverse Drug Reactions during Treatment

During the intervention, a total of six patients in the two groups developed new-onset gastrointestinal symptoms, which were assessed as adverse reactions related to PPI medications and none of them experienced serious adverse events (Table 2). In the Placebo group, one patient experienced nausea and vomiting, two patients experienced abdominal bloating, and two patients had diarrhea. While in the Probiotic group, only one patient experienced nausea and vomiting. There was no statistical difference in the incidence of adverse reactions between the two groups (1.96% vs. 10.20%, *p* = 0.108).

## 4. Discussion

Based on statistics from the global population, the prevalence of GERD varies from 8% to 33% between different countries, revealing it as a significant global health concern [2]. RE is an important manifestation of GERD, characterized not only by typical clinical symptoms such as acid regurgitation and heartburn, but also accompanied by symptoms like nausea, belching, upper abdominal pain, and bloating [28]. Finding ways to promptly relieve RE symptoms and reduce the frequency of recurrence has been a longstanding clinical challenge for clinicians. This study aimed to explore the potential benefits of supplementing with *Bifidobacterium animalis* subsp. *Lactis* MH-02 as an adjunctive treatment for RE. The results of this double-blind, randomized, placebo-controlled trial revealed positive therapeutic effects of probiotics in improving RE symptoms, delaying recurrence, and remodeling gut microecology.

Current research indicates that the key to treating RE is acid suppression therapy, which aims to reduce the erosive effects of gastric reflux on the esophageal mucosa by increasing the pH within the stomach. Proton pump inhibitors (PPIs) are presently the most widely used acid-suppressing medications, significantly alleviating acid regurgitation and heartburn symptoms in RE patients [4]. However, RE often includes additional symptoms like upper abdominal pain and bloating, necessitating other treatments, including prokinetic medications, which may also impose an extra burden on the body, particularly for patients with a history of cardiovascular conditions. Probiotics have shown promise in managing these symptoms. In a review of GERD and probiotics, 11 out of 13 selected studies reported beneficial effects of probiotics on reflux, dyspeptic symptoms, nausea, and abdominal discomfort [29]. Additionally, some clinical studies have demonstrated that probiotics can effectively promote gastric emptying and reduce reflux symptom in infants and pregnant women with functional gastrointestinal disorders [30,31]. As anticipated, our study demonstrated that the Probiotics intervention group, when compared to the Placebo group, exhibited a swifter relief of acid reflux and heartburn symptoms in the first 2 weeks of treatment. No differences in remission rate and cure rate of the main symptoms were observed during the 4 and 8 weeks of treatment, which were mainly attributed to the predominant therapeutic effect of rabeprazole over time. Furthermore, we observed that after 8 weeks of treatment, the Probiotic group had a significantly lower GSRS score than the Placebo group. Finally, the study also showed that the combination of MH-02 for RE did not increase adverse effects, and there seemed to be a trend to decrease. This suggests that MH-02 in combination with rabeprazole is safe and may be beneficial in achieving early pain relief, contributing to patient quality of life, and potentially improving patient compliance.

The frequent symptom recurrence of RE presents a significant challenge in treatment, often requiring prolonged use of PPIs medications for maintenance. Recent studies have linked long-term PPIs usage to issues like impaired nutrient absorption, bacterial translocation infections, and gut microbiota imbalance [32,33]. In this study, we conducted a 12-week follow-up on RE patients who had achieved remission after an 8-week treatment. The results yielded a promising discovery: MH-02 combined therapy can delay the time of recurrence compared to the conventional therapeutic regimen. This suggests that combining probiotics with conventional RE treatment may reduce the need for PPIs medication throughout the course of the disease, which can contribute to improved quality of life of patients and further reduce the medical burden. The precise mechanism by which probiotics influence the time of recurrence remains unclear. Prior researchers have suggested that small intestinal bacterial overgrowth (SIBO) may contribute to and worsen esophageal reflux through increased gas production, higher abdominal pressure, and activation of gastrointestinal immune–inflammatory pathways [34,35,36]. The acid suppression caused by PPIs is considered as a precursor to the development of SIBO. Tsuda et al. [37] found that PPIs application for just 4 weeks can lead to the development of SIBO in a clinical study of patients with functional dyspepsia. Jacobs et al. identified PPI use as an independent risk factor for SIBO, and meta-analyses support this link [38,39,40]. Conversely, multiple studies suggested that probiotics can ameliorate the symptoms and prognosis of SIBO [41,42,43]. Therefore, we speculate that the delay in RE recurrence attributed to probiotics may be partially associated with this effect. However, specific pathological and physiological mechanisms require further investigation in the future.

The gut microbiota and its metabolites are well known to exert an instrumental role in human health and diseases by modulating both body metabolism and immune function. However, research concerning the interplay between gut microbiota and RE still remains a huge gap in the present scientific community. A study by Shi et al. [6] suggested significant differences in the composition and abundance of gut microbiota between GERD patients and healthy individuals. Additionally, another recent report indicated that microbiota transplantation therapy can greatly elevate overall remission rate in patients with non-erosive gastroesophageal reflux, implying that restoration of normal gut microbiota may play a crucial role in managing GRED [44]. Thus, in order to further explore the underlying mechanism of MH-02-mediated improvement of RE, we conducted high-throughput sequencing on fecal samples of patients, which demonstrated that co-administration of probiotics increased the α-diversity of gut microbiota and altered microbial composition by β-diversity analysis in patients with RE. However, no significant difference in α-diversity was observed between the RE and Placebo groups, and there was a mild separation of sample clusters in terms of β-diversity, suggesting that the use of proton pump inhibitors (PPI) in this study only slightly affect the diversity of the intestinal microbiota in the patient cohort. These alterations are consistent with previous studies [6,45,46]. On the other hand, this implies that co-administration of probiotics therapy may drive the intestinal microbiota of patients towards a healthier profile, potentially enhancing RE management.

Further analysis on the composition of the intestinal microbiota, which is presented in Figure 5, demonstrated significant changes in the abundance of taxa at the genus level following the co-administration of PPI with MH-02 treatment. The clustering heatmap and LEfSe analysis provided a more intuitive representation of the inter-group variations in bacterial abundance that, compared to the Placebo group, the Probio group exhibited an enrichment of taxa belonging to *Bifidobacterium*, *Clostridiaceae_Clostridium*, *Blautia*, *Coprococcus*, and *Phascolarctobacterium* genera, suggesting an improvement in intestinal microecology. Mounting evidence supports the role of *Bifidobacterium* in supporting host immune system development, improving intestinal homeostasis and function, and preventing pathogen proliferation [17,47,48]. Additionally, *Bifidobacterium* can produce beneficial metabolites like short-chain fatty acids, which are regarded to have positive effects on host epithelial cells and gut microbiota [49,50,51]. Further, *Clostridiaceae_Clostridium* contains a group of beneficial bacteria including *C. butyrate* that can produce butyrate, which has the capacity to significantly lower the pH within the intestinal tract and can effectively promote the growth of normal gut microbiota, including *Bifidobacteria* [52,53]. The *Blautia* genus may also play a beneficial role in metabolic diseases, inflammatory diseases, and biotransformation [54,55,56,57]. In contrast, relative to the Placebo group, the Probio group exhibited a significant decrease in the abundance of *Streptococcus*, *Lactobacillus*, *Rothia*, and *Sutterella*. Some previous studies have reported a significant increase in *Streptococcus* abundance in the gut after PPIs use in patients, including GERD patients [11,26,32]. In the current study, we seem to have reversed this increase after using MH-02. Although *Streptococcus* is one of the common genera of normal esophageal microorganisms [58], when gastric acid in the stomach is inhibited, and *Streptococcus* enters the lower digestive tract, it is often associated with some disease states [26,59,60]. This also suggests that *Streptococcus* has different distributions and functions at different sites [61]. Interestingly, *Lactobacillus*, which is generally considered beneficial, had a higher abundance in the Placebo group and a lower abundance in the Probio group. On the other hand, some studies reported increased *Lactobacillus* abundance in esophageal disease, but most of these were considered to be related to the PPIs. The long-term utilization of PPIs induces gut dysbiosis by modifying the composition and diversity of the gut microbiota, commonly resulting in an elevated abundance of *Streptococcus* and *Lactobacillus* [11,62]. However, it is not clear how the elevation of *Lactobacillus* after PPIs use affects the body. There are also studies that have reported serious infections due to *Lactobacillus*, such as bacteremia and liver abscess [63,64], and an increase in *Lactobacillus* has also been observed in patients with diseases such as type 2 diabetes and Parkinson’s disease [65,66]. This may indicate that some bacteria need to maintain appropriate concentrations for their beneficial effects, and that the coevolutionary relationship between the bacteria and their hosts determines the characteristics of probiotics [67,68]. Therefore, the supplementation of MH-02 in this study may maintain *Lactobacillus* homeostasis, but this needs to be confirmed by more studies. Rothia also has been identified as a common opportunistic pathogen in human body, which can cause tonsillitis, pneumonia, and endocarditis [61,69]. And, *Sutterella* has also been reported to be associated with ulcerative colitis, antibiotic-associated diarrhea, and other gastrointestinal diseases [70,71]. Meanwhile, in this trail, probiotics intervention can counteract the changes observed in these microbial populations, and moreover contribute to the proliferation of beneficial microbiota and prevent the growth of potential pathogens. In summary, in view of these findings, it strongly indicates that supplementation of probiotics in treating RE can reshape the gut microbiota, favoring a more favorable balance in the microecology.

Currently, there is a paucity of high-quality clinical research on the application of probiotics as an adjunct therapy for RE. This study provides valuable references for future research in this direction. However, this study also has certain limitations. Firstly, although efforts were made to exclude drugs or foods containing probiotics and prebiotics that may interfere with the trial, it was not possible to standardize dietary intake completely, resulting in potential variations in diet-induced gut microbiota. Secondly, although we have analyzed improvements in clinical symptoms and the intestinal microbiota, direct evidence linking the two has not yet been explored. A recent study by Liu et al. [10] performed proteomic analysis to investigate the mechanisms by which microbiota mediated esophageal injury in RE, suggested that the imbalance in esophageal and gut microbiota can elicit ferroptosis and pyroptosis by increasing circulating LPS levels. Therefore, we hypothesize that supplementing MH-02 may act through remolding gut microbiota, regulating LPS levels and other microbial metabolic products, to ultimately reduce esophageal mucosal damage and promote mucosal repair. Further research investigating the mechanisms by which probiotics mediate these improvements will aid in a deeper understanding of the potential therapeutic effect of probiotics in RE. Further, our sample size and follow-up time were limited, and grade stratification of reflux esophagitis was not carried out for respective observation and comparison. Therefore, in the future, larger cohorts and longer periods are needed to explore the continuous benefits of probiotics in RE.

## 5. Conclusions

**Figure 6:**
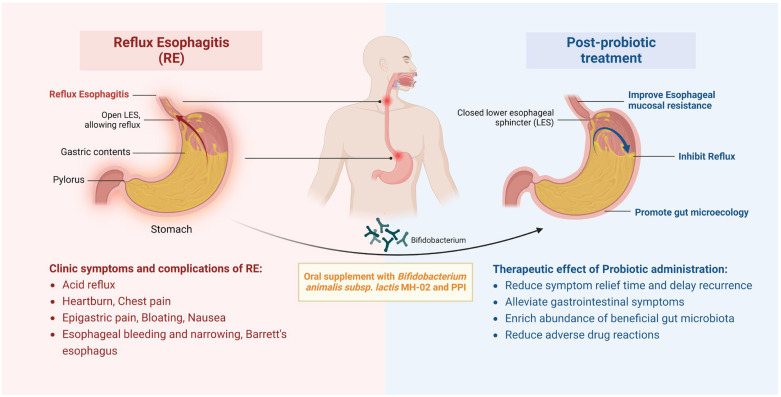
**Schematic of therapeutic improvements by administrating MH-02 and PPI for RE.** Oral supplement with *Bifidobacterium animalis* subsp. *lactis* MH−02 together with PPI can contribute to inhibit acid reflux, alleviate gastrointestinal symptoms, and reduce symptom relief time and delay recurrence, enrich gastrointestinal beneficial microbiota, and reduce adverse drug reactions.

In conclusion, the results of this study suggest that combined MH-02 supplementation can assist in improving the treatment outcomes of RE patients, including early relief of typical symptoms, improvement of associated gastrointestinal symptoms, and delayed recurrence (Figure 6). Moreover, MH-02 may help restore the balance of the gut microbiota, with changes in certain bacterial genera potentially explaining some of its effects. Thus, this represents a promising new treatment strategy to improve the quality of life of RE patients and promote gut health, offering a more comprehensive and potentially more effective approach for clinicians to manage this chronic condition in the future.

## Figures and Tables

**Table 1:** Baseline characteristics of patients.

Characteristic		Probiotics Group (n = 51)	Placebo Group (n = 49)	*p*-Value
Age (y) (mean ± SD)		50.80 ± 7.69	52.65 ± 7.65	0.231
Sex (male, n [%])		27 (52.94)	28 (57.14)	0.693
BMI (kg/m^2^)		24.63 ± 3.27	24.01 ± 3.26	0.349
Smoking (n, [%])		17 (33.33)	19 (38.78)	0.678
Anxiety–depression tendency (n, [%])		18 (35.29)	15 (30.61)	0.674
RDQ score (mean ± SD)		19.14 ± 5.66	20.22 ± 5.37	0.327
GSRS score (median [25%,75%])		30 (22, 33)	29 (26, 32)	0.565
Esophagitis grade (n)	A	13	15	0.849
	B	27	24	
	C	11	10	

**Table 2:** Summary of adverse events.

Adverse Events	Probiotics Group (n = 51)	Placebo Group (n = 49)	*p*-Value
Number of patients with adverse events (n [%])	1 (1.96%)	5 (10.20%)	0.108
Nausea and vomiting	1	1	
Abdominal bloating	0	2	
Diarrhea	0	2	

## Data Availability

The raw data of 16S rRNA high-throughput sequencing was uploaded to the Sequence Read Archive (SRA) database of NCBI (PRJNA1036777). Other data will be made available by the authors, without undue reservation.

## Supplementary Materials

The following supporting information can be downloaded at: https://www.mdpi.com/article/10.3390/nu16030342/s1, Supplementary Figure S1: Changes in the diversity of the gut microbiota, **: *p* < 0.01; Supplementary Figure S2: Relative abundance of bacterium at the phylum level; Supplementary Figure S3: LEfSe cladogram showing differently abundant gut microbiota taxa among RE patients at different levels.
