# Supplementary figures and images for: Therapeutic Evaluation of Bifidobacterium animalis subsp. lactis MH-02 as an Adjunctive Treatment in Patients with Reflux Esophagitis: A Randomized, Double-Blind, Placebo-Controlled Trial
Source: Nutrients. 2024 Jan 24;16(3):342. doi: 10.3390/nu16030342 (PMC10856834; doi:10.3390/nu16030342)

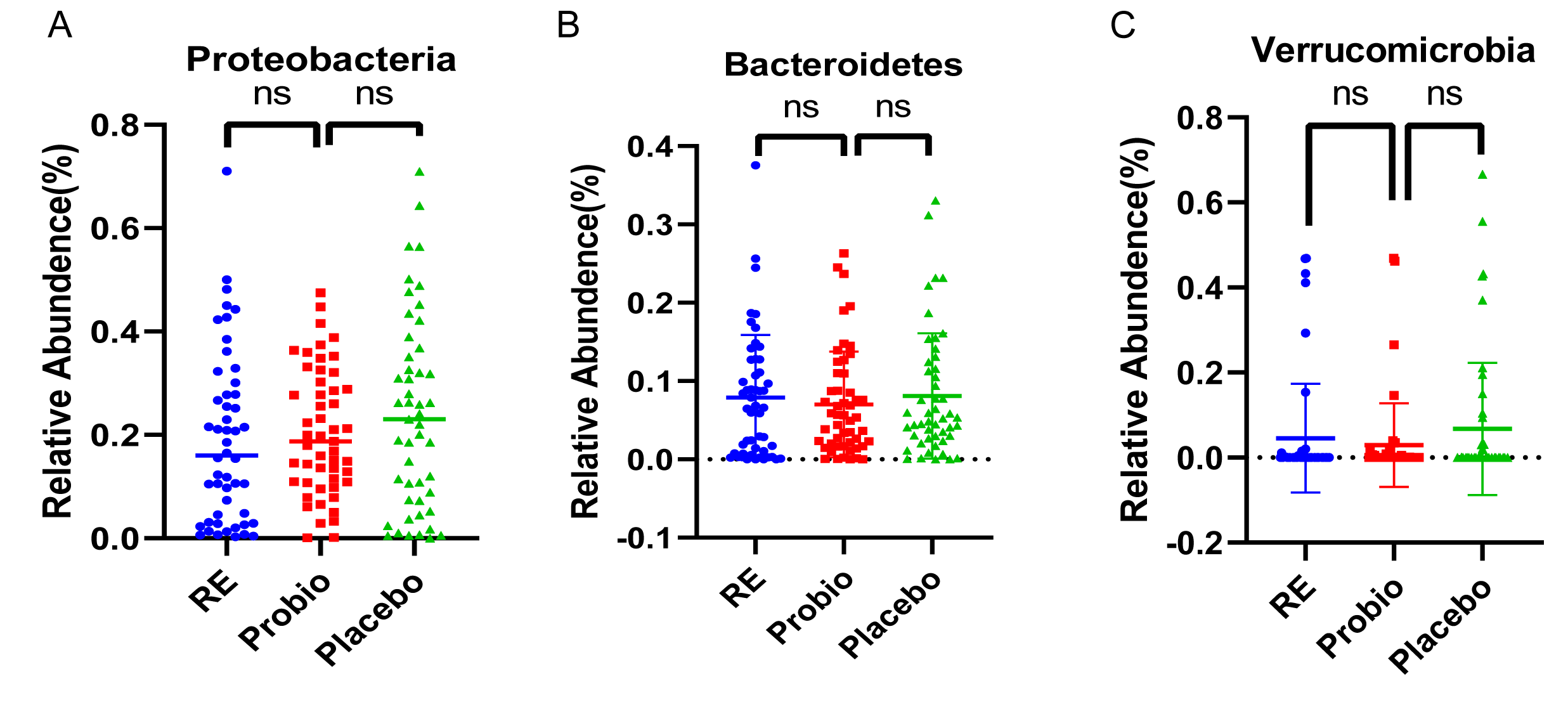

Supplement: Supplementary file 1 [file nutrients-16-00342-s001.zip › Supplement Figure S2.tif]
